# Health multidimensional evaluation of institutionalized older adults according to cognitive performance

**DOI:** 10.1590/1980-5764-DN-2024-0133

**Published:** 2025-04-07

**Authors:** Beatriz Cintra Martins, Lilian Barbosa Ramos, Anna Karla Carneiro Roriz, Henrique Salmazo da Silva

**Affiliations:** 1 Universidade Católica de Brasília, Brasília DF, Brazil.; 2 Universidade Federal da Bahia, Pós-Graduação em Alimento, Nutrição e Saúde, Salvador BA, Brazil.; 3 Universidade Católica de Brasília, Programa de Graduação em Gerontologia, Brasília DF, Brazil.

**Keywords:** Aged, Homes for the Aged, Health Status, Neuropsychological Tests, Geriatrics, Idoso, Instituição de Longa Permanência para Idosos, Nível de Saúde, Testes Neuropsicológicos, Geriatria

## Abstract

The assessment of living and health conditions of the institutionalized older adults contributes to the development of health promotion measures. The high prevalence of cognitive decline in this population leads to unfavorable health outcomes, which can be prevented and managed.

**Objective:**

To investigate the health conditions of institutionalized older adults according to global cognitive performance.

**Methods:**

Cross-sectional study of institutionalized older adults in Brasília, Distrito Federal (DF) and Salvador, Bahia (BA) using questionnaires on multidimensional health assessment and the Mini-Mental State Examination (MMSE).

**Results:**

Of the 185 residents, 86 were evaluated, with the majority of the sample composed of women and people aged 80 years old or older. Cognitive decline in the MMSE was associated with greater difficulties in Activities of Daily Living (ADL) (62.5%). Lower performance on the MMSE was correlated with longer length of institutionalization (p=0.043), older age (p=0.004), poorer Geriatric Oral Health Assessment Index (GOHAI) (p=0.087), greater difficulty in ADL (p<0.001), and higher scores on the Bristol scale (p<0.001). Conversely, higher education (p<0.001), better scores on the Mini Nutritional Assessment (p=0.003), larger Calf Circumference (CC) (p=0.036), and Hand Grip Strength (HGS) (p=0.0467) were associated with better performance in the MMSE.

**Conclusion:**

The cognitive performance of institutionalized older people is correlated with sociodemographic and health variables. These results are oriented toward the formulation of public health policies and care management.

## RESUMO.

A avaliação das condições de vida e saúde da população idosa institucionalizada contribui para o delineamento de medidas de promoção de saúde. A elevada prevalência de declínio cognitivo nesta população cursa com desfechos de saúde desfavoráveis, que podem ser prevenidos e gerenciados.

**Objetivo:**

Investigar as condições de saúde das pessoas idosas institucionalizadas conforme o desempenho cognitivo global.

**Métodos:**

Estudo transversal em pessoas idosas de Brasília/DF e Salvador/BA por meio de questionários sobre avaliação multidimensional da saúde e o Miniexame do Estado Mental (MEEM).

**Resultados:**

De 185 residentes, avaliou-se 86, sendo a maioria da amostra composta por mulheres e pessoas com 80 anos ou mais. Declínio cognitivo no MEEM foi associado com maiores dificuldades nas Atividades Básicas de Vida Diária (ABVD) (62.5%). Observou-se que quanto menor o desempenho no MEEM, maior o tempo de permanência na instituição (p=0.043), maior a idade (p=0.004), pior o Índice de Avaliação da Saúde Bucal Geriátrica (GOHAI) (p=0.087), maior o número de dificuldades nas ABVD (p<0.001) e maior o escore na escala Bristol (p<0.001). Inversamente, maior escolaridade (p<0.001), melhor pontuação na Mini Avaliação Nutricional (p=0.003), maiores medidas de Circunferência de Panturrilha (CP) (p=0.036) e força de Preensão Manual (FPM) (p=0.467) se associaram ao melhor desempenho no MEEM.

**Conclusão:**

O desempenho cognitivo de pessoas idosas institucionalizadas se correlaciona com variáveis sociodemográficas e saúde. Os resultados orientam-se para formulação de políticas públicas de saúde e de gestão da atenção.

## INTRODUCTION

The unprecedented increase in the older population challenges social and healthcare systems. According to recent projections, the older adult population in Brazil will triple, rising from 19.6 million in 2010 to 66.5 million by 2050, becoming the sixth-largest older adult population in the world^1^.

The definition of health is not just a theoretical issue, as it has many implications for health practice, policies, and services. According to the World Health Organization (WHO), health is a state of complete physical, mental, and social well-being, rather than merely the absence of disease or infirmity. Currently, a multifaceted concept of health is addressed, extending beyond the absence of disease. Health is defined as the experience of physical and psychological well-being, incorporating a holistic well-being^2^.

Aging follows different trajectories, often involving a gradual reduction in functionality, which can lead to physical and/or cognitive dependence, associated with an increased demand for long-term care and continued care services. Multidimensional assessment is essential for evaluating the health and needs of older adults, particularly those experiencing cognitive decline^3^. It is estimated that approximately 60% of the older adults living in Long-Term Care Facilities (LTCF) exhibit cognitive decline^4^, which demands the development of tools, assessments, and care strategies tailored to this population.

Cognitive decline is characterized by the impairment of important skills, such as memory, attention, visuospatial ability, language, and executive functions^5^, and often precedes more severe diagnoses, in particular Alzheimer disease or other dementias. Maintaining multidimensional cognitive ability is related to longevity, satisfactory quality of life, and physical and social well-being^6^. Furthermore, individual demographic and socioeconomic factors, such as age, gender, education, marital status, and self-perceived health, are also strongly associated with cognitive function^7^.

The functional status of older adults is commonly assessed through their performance in activities of daily living (ADLs), which are didactically divided into three groups:

- Basic: everyday tasks directly related to survival;
- Instrumental: tasks involved in maintaining community life; and
- Advanced: more complex activities, subdivided into physical, leisure, social, and productive domains^8^.

The loss of independence and functional incapacity among older adults, combined with the lack of social assistance and public policies for the long-lived population, are factors that lead to institutionalization^9^. According to Fagundes et al.^4^, most residents in LTCFs have a high degree of cognitive and physical impairment. However, in Brazil, institutionalization may result in a lack of resources for the development of comprehensive care. Many Brazilian institutions operate as philanthropic organizations, and the Unified Health System (SUS) lacks programmatic frameworks specifically designed to serve the needs of this demographic.

To discuss the health indicators of LTCF residents in Brazil, several factors influencing their well-being must be considered. Research shows that LTCF residents in Brazil have faced challenges related to COVID-19, including high mortality rates among residents^10^. The COVID-19 pandemic has further emphasized vulnerabilities in LTCFs in Brazil, such as insufficient pandemic preparedness and resources, impacting residents’ health outcomes^11^.

Current research on the health of institutionalized older people has focused on multifaceted aspects that influence their quality of life, with an emphasis on factors such as fall prevention, chronic disease management, mental health, and the impact of institutionalization on overall well-being. Studies have highlighted the importance of integrated and personalized care approaches for these populations, who often face greater vulnerability due to physical, cognitive, and emotional conditions^12^. In addition, research points to the need for rehabilitation programs and activities that promote autonomy and socialization, essential elements to improve health status and reduce the progression of functional decline.

Despite numerous studies addressing cognitive decline in institutionalized older adults, few Brazilian studies have evaluated the multidimensional health conditions of older people according to cognitive performance. Health indicators among LTCF residents in Brazil encompass various factors, including mental health, healthcare strategies and disparities. Addressing these indicators comprehensively and implementing targeted interventions can enhance both the overall well-being and the quality of care of LTCF residents in Brazil. Mapping these conditions is the first step toward establishing prevention, promotion, and comprehensive health care measures. This approach contributes to the planning of health policies tailored to the unique needs of this population. Given that a substantial portion of institutionalized older adults experience cognitive and functional decline, this study contributes to the design of better models for multidimensional assessments. These data enable the development of multimodal and comprehensive interventions, addressing the lack of information on the part of professionals. The objective of the present study was to investigate the health conditions of institutionalized older adults according to global cognitive performance in the Mini Mental State Examination (MMSE).

## METHODS

### Study design and participants

This is a quantitative, cross-sectional, and observational research linked to the Postgraduate Program in Gerontology at *Universidade Católica de Brasília* and the Postgraduate Program in Food, Nutrition, and Health at *Universidade Federal da Bahia* (UFBA).

Three nursing homes participated in the study: one located in the city of Brasília, Brazil, and two in the city of Salvador, Brazil. Of the initial total sample of 185 residents (90 from Brasília and 95 from Salvador), only 86 were ultimately included in the study. The loss of potential participants was higher in Brasília: of 90 older adults, 70 were potentially eligible, but only 22 were investigated. In Salvador, of 95 participants, a total of 75 were deemed eligible, and 64 were investigated. The study was initiated in January but had to be interrupted in March due to the Covid-19 pandemic.

The three participating nursing homes were selected based on convenience and the following inclusion criteria:

- Being public or philanthropic;
- Operating in the same physical location for at least two years;
- Having a technical manager available to participate in study activities;
- Providing consent to participate in the study.

In both cities, four institutions met all the inclusion criteria. Two institutions from Brasília and three from Salvador provided informed consent. However, due to limitations imposed by the Covid-19 pandemic, only one institution in Brasília and two in Salvador were included.

As for the inclusion criteria, participants were required to have lived in the nursing home for at least six months and had to agree to participate in the study. There were no restrictions on age and gender. Exclusion criteria included sensory impairments or difficulties understanding the questions; aphasia, agnosia, or speech and language impairments that could hinder communication; and bedridden individuals or individuals with untreated psychiatric morbidities. Medical records were reviewed to identify sensory and psychiatric difficulties. These criteria were employed due to the self-report nature of the questions used for the evaluations.

The exclusion criterion of untreated psychiatric disorders was adopted because the clinical stage and behavioral symptoms were not assessed. However, although no evaluation of possible behavioral disorders was performed, the team analyzed medical records and only evaluated aged people with medication follow-up or without reports of behavioral complaints. Of the 46 participants with cognitive decline on the MMSE, 43 were on continuous medication. These included anxiolytics, antidepressants, and antipsychotics, such as: risperidone (n=9), sertraline (n=1), haloperidol (n=1), clonazepam (n=5), carbamazepine (n=2), citalopram (n=1), fluoxetine (n=1), escitalopram (n=1), valproic acid (n=4), amitriptyline (n=3), olanzapine (n=1), pericyazine (n=1), zolpidem (n=1), quetiapine (n=1), and diazepam (n=1).

### Procedures

The evaluations were carried out between January and March 2020, prior to the COVID-19 Pandemic was decreed in Brazil, with the participation of a team of 15 undergraduate and postgraduate students from each research center. To carry out the evaluations, all participants were informed about the objectives of the study and, subsequently, signed the Informed Consent Form (ICF), in compliance with the ethical principles of research in accordance with Ordinance 466/2012 of the Ministry of Health (MoH).

This study was approved by the Research Ethics Committee of *Universidade Católica de Brasília* (CAAE: 18151019.1.1001.0029, technical advice: 3.621.190) and the Ethics Committee of the School of Nutrition of *Universidade Federal da Bahia* (CAAE: 18561419.5.1001.5023, technical advice: 3.793.529).

### Instruments and variables

For multidimensional health assessment and sociodemographic characterization of participants, the instruments applied were:

- The sociodemographic questionnaire, prepared by the researchers, with information on gender, age, education, marital status, and length of institutionalization.
- Health questionnaire, prepared by the researchers, with information on the interviewee's general health status, polypharmacy (five or more medications), number of self-reported chronic diseases, presence of urinary incontinence, and loss of appetite.
- MMSE questionnaire is composed of questions that assess attention skills, memory, orientation, comprehension, and naming^13^. The cutoff points adopted in the present study were: 13 for illiterate; 18 for people with 1 to 8 years of education; and 26 for people with 9 or more years of schooling^14^. Sensitivity for these cutoff points were 82.4% for illiterate individuals, 75.6% for 1 to 8 years of study, and 80% for high schooling (9 years and more); and specificity of 97.5%, 96.6%, and 95.6%, respectively^14^. In the present study, the MMSE was used as the dependent variable, with participants classified into two groups: presence or absence of cognitive decline. Additionally, the MMSE was used as a discrete quantitative variable, whose score ranged from 0 to 30 points.
- Katz's functional performance questionnaire in ADLs: absence or presence of difficulties in ADLs^15^, including self-care tasks such as using the bathroom, eating, mobility, transferring, bathing, and personal hygiene. Participants were classified as either "without limitations" or "with one or more limitations."
- Mini Nutritional Assessment (MNA): scores range from 0 to 30, based on the sum of the values of 18 items regarding the anthropometric, global, dietary, and subjective assessment dimensions. A score equal to or greater than 24 indicated good nutritional status, scores between 17 and 23.5 indicates a risk of malnutrition, and scores below 17 identified patients with protein-calorie malnutrition^16^. Criterion validity was also evidenced with relevant sensitivity (82.8) and specificity (80.0).
- Anthropometric assessment: included body weight and height, and waist (WC) and calf (CC) circumferences.
- Bristol Stool Form scale for stool description^17^.
- Geriatric Oral Health Assessment Index (GOHAI): consisted of 12 questions assessing the quality of life of older adults in relation to oral health across three domains: physical function (eating, speaking, and swallowing), psychosocial factors (dissatisfaction with appearance and socialization), and painful symptoms^18^. The index categorizes self-perception into three groups: poor (0 to 30 points), regular (31 to 33 points), or excellent (34 to 36 points).

### Data analysis

The data were analyzed descriptively and inferentially using the JAMOVI 2.3.24 Program. Performance on the MMSE was treated as a categorical variable (with or without decline according to the Bertolucci et al.^14^ cutoff point) and a discrete quantitative variable (number of points on the MMSE, up to 30 points). The χ^2^ test was applied to categorical variables, followed by post-hoc analyses of those with statistical significance. The Spearman correlation test was used to analyze the MMSE as a discrete quantitative variable. Statistical significance was set at p<0.05.

## RESULTS

Of the 86 institutionalized older adults investigated, an equivalent distribution was observed (around 30%) across the age groups of 70 to 79 years, 80 to 89 years, and 90 years old and older, with the age group of 60 to 69 years being less prevalent (20.9%). The average age of participants was 79.3 years (standard deviation=10.3). Most participants were female (60.5%), had between 5 and 8 years of education (33.7%), with an average of 6.5 years of schooling, and declared themselves without a partner (48.2% single, 22.4% divorced, and 20% widowed) (Table 1).

**Table 1:** Characterization of social profile and the health conditions of institutionalized aged people in Salvador (BA) and Brasília (DF), 2020.

Characteristics		n	%
Age (years)			
	60 to 69	18	20.9
	70 to 79	26	30.2
	80 to 89	25	29.1
	90 and more	26	30.2
Gender			
	Female	52	60.5
	Male	34	39.5
Education (years)			
	Illiterate	15	18.1
	1 to 4	18	21.7
	5 to 8	28	33.7
	9 and more	22	26.5
Marital status			
	Married/Partner	8	9.4
	Single	41	48.2
	Divorced/Separated	19	22.4
	Widow/Widower	17	20.0
MMSE cognitive decline			
	No decline	39	45.9
	Cognitive decline	46	54.1
Polypharmacy (medications)			
	0 to 4	38	50.0
	5 or more	38	50.0
GOHAI Index (points)			
	Poor (0 to 30)	49	73.1
	Regular (30 to 33)	13	19.4
	Excellent (34 to 36)	5	7.5
ADL			
	No difficulties	45	57.0
	1 limitation or more	34	43.0
Urinary incontinence			
	No	59	71.1
	Yes	24	28.9
Fecal incontinence			
	No	74	90.2
	Yes	8	9.8
Loss of appetite			
	No	57	67.1
	Yes	28	32.9
Hospitalization			
	No	62	74.7
	Yes	21	25.3
Difficulty chewing			
	No	59	69.4
	Yes	26	30.6
Hypertension			
	No	29	33.7
	Yes	57	66.3
Stroke			
	No	71	82.6
	Yes	15	17.4

Abbreviations: MMSE, Mini-Mental State Examination; GOHAI, Geriatric Oral Health Assessment Index; ADL, Activities of Daily Living.

Regarding health status (Table 1), 54.1% of participants exhibited cognitive decline based on the MMSE. High blood pressure (HBP) and limitations in ADL were the most prevalent health conditions in the sample investigated.

Table 2 shows that the groups with and without cognitive decline had a similar health profile overall. However, the group with cognitive decline had a higher prevalence of difficulties in ADL (62.5%) when compared to the cognitively healthy group (23.7%) (p<0.01).

**Table 2:** Cognitive decline in institutionalized aged people according to sociodemographic and health conditions, Salvador (BA) and Brasília (DF), 2020.

Characteristics		Without MMSE cognitive decline	With MMSE cognitive decline	p-value*
		n (%) (95%CI)	n (%) (95%CI)	
Gender				
	Female	21 (53.8) (37–69)	31 (67.4) (51–80)	0.265
	Male	18 (46.2) (30–62)	15 (32.6) (19–48)	
Age (years)				
	60 to 69	8 (20.5) (9–36)	10 (21.7) (10–36)	0.361
	70 to 79	15 (38.5) (23–55)	11 (23.9) (12–38)	
	80 to 89	11 (28.2) (15–44)	13 (28.3) (15–43)	
	90 and more	5 (12.8) (4–27)	12 (26.1) (14–41)	
Marital status				
	Married/Partner	3 (7.7) (1–20)	4 (8.9) (2–21)	0.805
	Single	20 (51.3) (34–67)	21 (46.7) (31–62)	
	Divorced/Separated	7 (17.9) (7–33)	12 (26.7) (14–41)	
	Widow/Widower	9 (23.1) (11–39)	8 (17.8) (8–32)	
Education (years)				
	Illiterate	7(17.9) (7–33)	8 (18.2) (8–32)	0.381
	1 to 4	10 (25.6) (13–42)	8 (18.2) (8–32)	
	5 to 8	15 (38.5) (23–55)	13 (29.5) (16–45)	
	9 and more	7 (17.9) (7–33)	15 (34.1) (20–49)	
Polypharmacy (medications)				
	0 to 4	15 (45.4) (28–63)	23 (53.5) (37–68)	0.644
	5 or more	18 (54.5) (36–71)	20 (46.5) (31–62)	
GOHAI Index (points)				
	Poor (0 to 30)	28 (75.7) (58–88)	20 (69.0) (49–84)	0.722
	Regular (30 to 33)	6 (16.2) (6–32)	7 (24.1) (10–43)	
	Excellent (34 to 36)	3 (8.1) (1–21)	2 (6.9) (0.1–22)	
ADL				
	No difficulties	29 (76.3) (59–88)	15 (37.5) (22–54)	<0.001
	1 limitation or more	9 (23.7) (11–40)	25 (62.5) (45–77)	
Urinary incontinence				
	No	28 (71.8) (55–85)	30 (69.8) (53–82)	0.840
	Yes	11 (28.2) (15–44)	13 (30.2) (17–46)	
Fecal incontinence				
	No	35 (92.1) (78–98)	38 (88.4) (74–96)	0.717
	Yes	3 (7.9) (1–21)	5 (11.6) (3–25)	
Loss of appetite				
	No	27 (69.2) (52–83)	29 (64.4) (48–78)	0.643
	Yes	12 (30.8) (17–47)	16 (35.6) (21–51)	
Hospitalization				
	No	30 (76.9) (60–88)	31 (72.1) (56–84)	0.616
	Yes	9 (23.1) (11–39)	12 (27.9) (15–43)	
Difficulty chewing				
	No	27 (69.2) (52–83)	31 (68.9) (53–81)	0.973
	Yes	12 (30.8) (17–47)	14 (31.1) (18–46)	
Hypertension				
	No	13 (33.3) (19–50)	15 (32.6) (19–48)	0.944
	Yes	26 (66.7) (49–80)	31 (67.4) (51–80)	
Stroke				
	No	34 (87.2) (72–95)	36 (78.3) (63–89)	0.394
	Yes	5 (12.8) (4–27)	10 (21.7) (10–36)	

Abbreviations: MMSE: Mini-Mental State Examination; GOHAI: Geriatric Oral Health Assessment Index; ADL: Activities of Daily Living.

Notes:

* χ^2^.

When the analyses considered the total MMSE score (Table 3), without stratifying the cutoff point for cognitive decline, it was found that poorer MMSE scores were observed in women and those with lower educational attainment. There was an association between the MMSE score and the GOHAI index, but the differences were not evident in post-hoc analyses. The correlation test did not demonstrate statistical significance between the MMSE score and other variables.

**Table 3:** Mini-Mental State Examination scores in institutionalized aged people according to sociodemographic and categorical health variables, Brasília (DF) and Salvador (BA), 2020.

Characteristics		n	Mean	Median	95%CI	SD	p-value
Gender							
	Female	52	17.4	18.0	15.6–19.2	6.35	0.026
	Male	34	20.2	21.0	18.1–22.3	6.04	
Education* (years)							
	Illiterate	15	12.4	12.0	9.86–14.9	4.58	<0.001
	1 to 4	18	17.6	18.5	15.04–20.1	5.06	
	5 to 8	28	19.5	20.0	17.46–21.5	5.25	
	9 and more	22	23.5	24.0	21.63–25.5	4.32	
Marital status							
	Married/Partner	8	18.3	18.5	12.8–23.7	6.50	0.606
	Single	41	17.8	19.0	15.8–19.8	6.34	
	Divorced/Separated	19	19.2	21.0	16.6–21.8	5.40	
	Widow/Widower	17	19.8	20.0	15.9–23.6	7.47	
Polypharmacy (medications)							
	0 to 4	38	19.5	20.5	17.3–21.7	6.67	0.195
	5 or more	38	17.7	18.5	15.8–19.7	6.01	
GOHAI Index^†^ (points)							
	Poor (0 to 30)	49	19.0	19.0	17.7–20.4	4.74	0.044
	Regular (30 to 33)	13	20.8	24.0	16.3–25.4	7.57	
	Excellent (34 to 36)	5	23.8	24.0	18.0–29.6	4.66	

Abbreviations: GOHAI, Geriatric Oral Health Assessment Index; CI, confidence interval; SD, standard deviation.

Notes:

* post-hoc: Iliterates <1 a 4 years of schooling (p=0.025); literates <5 a 8 years of schooling (p<0.001); literates <9 years of schooling (p<0.001); 1 a 4 years of schooling <5 a 8 years of schooling (p=0.450); 1 a 4 years of schooling <9 years of schooling (p=0.004); 5 a 8 years of schooling <9 years of schooling (p=0.030); †post-hoc without statistical significance.

According to Table 4, positive correlations were observed between the MMSE score and the MNA score (rho=0.398; p=0.003), CC (rho=0.261; p=0.036), Right- and Left-Hand Grip Strength (rho=0.084; p=0.467). On the other hand, the MMSE score had a negative correlation with the time of institutionalization (rho=-0.221; p=0.043), age (rho=-0.309; p=0.004), GOHAI index (rho=0.211; p=0.087), number of difficulties in ADL (rho=-0.432; p<0.001), and Bristol Scale (rho=-0.524; p<0.001). The other data were not correlated with performance on the MMSE. Together, these data indicated that MMSE score were associated with anthropometric measures, functionality, nutritional status, oral health, and age.

**Table 4:** Correlations between performance on the Mini-Mental State Examination and sociodemographic and health variables.

Characteristics	Spearman's value (rho)	p-value
Time of Institutionalization	-0.221	0.043
Age	-0.309	0.004
GOHAI Index	0.211	0.087
Number of Difficulties in ADL	-0.432	<0.001
Bristol Scale Score	-0.524	<0.001
Education		<0.001
Mini Nutritional Assessment	0.398	0.003
Calf Circumference	0.261	0.036
Right- and Left-Hand Grip Strength	0.084	0.467

Abbreviations: GOHA,: Geriatric Oral Health Assessment Index; ADL, Activities of Daily Living.

**Figure 1:**
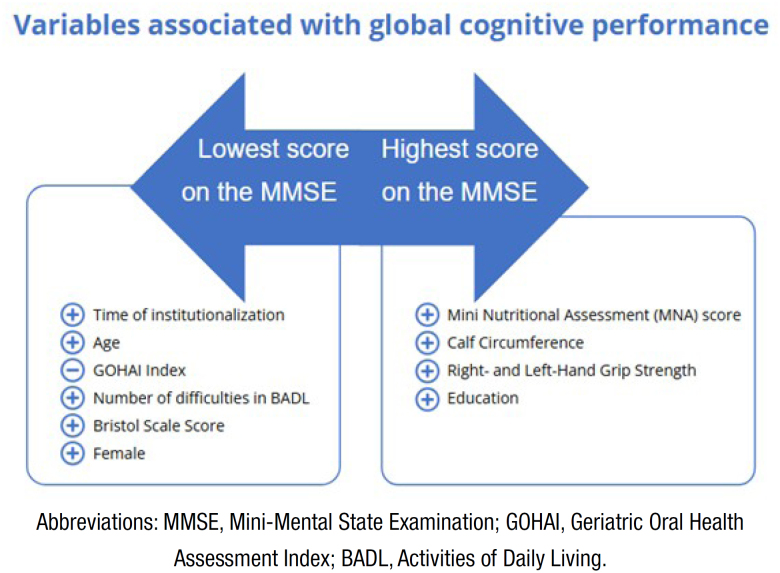
Summary of results.

Abbreviations: MMSE, Mini-Mental State Examination; GOHAI, Geriatric Oral Health Assessment Index; BADL, Activities of Daily Living.

In summary (Figure 1), lower MMSE performance was associated with longer length of institutionalization, older age, worse GOHAI scores, a greater number of ADL difficulties, higher Bristol scale scores, and a higher prevalence in females. Conversely, better MNA score, greater CC, stronger Hand Grip Strength (HGS), and higher education were associated with better performance in the MMSE.

## DISCUSSION

In the present study, participants with cognitive decline had a higher prevalence of difficulties in ADL. Additionally, when MMSE scores were analyzed, global cognitive status of institutionalized older adults were associated with anthropometric measures, nutritional status, functionality, oral health, and age. Therefore, these data indicate that the global cognitive performance of institutionalized older adults is related to multidimensional health indicators, that can be examined in greater depth and with greater interest by managers and professionals in nursing homes. It is important to highlight that participants with severe cognitive decline were excluded from the evaluation, that is, those who were unable to communicate or who had severe dementia.

While the data provide meaningful associations, they do not establish cause-and-effect relationships. Therefore, the data is exploratory and based on a small, non-probabilistic sample, which does not allow generalizations. Conducting a study with older adults living in nursing homes in Brazil is challenging due to several reasons: obtaining research authorization from institutions; securing funding to conduct the research; ensuring the availability of the teams to assist researchers and the availability of participants; using instruments with high sensitivity to correctly assess the health conditions of older adults living in nursing homes; and establishing evaluation schedules that do not disrupt the activities of the institutions. In addition, the Covid-19 pandemic significantly impacted the conduct of this study. For all these reasons, the study represents the collaborative efforts of the research team to describe exploratory data on cognitive decline.

In practical pathways, intervening in nutritional, anthropometric, functional, and oral health-related factors among institutionalized older adults requires the involvement of interprofessional health teams working and improvements in health care quality, with an interface with the primary, secondary, tertiary and quaternary health systems^19,20^. Some Brazilian institutions, in order to counter the more fragile profile of older adults individuals with cognitive decline, use dementia or mild cognitive decline as a criteria for non-admission^21^. This practice is especially observed in philanthropic institutions, whose prevalence is around 30–40% nationwide^22^. These institutions are believed to exclude participants with lower cognitive performance due to fewer resources and a worse interface with health care services.

This seems contradictory, given the high prevalence of aged people with some form of cognitive decline living in institutions, reaching around 70% of residents^23^. Due to the high daily healthcare costs for institutionalized older adults with dementia, in order of €151 for small-scale living wards and €147 for independent living wards^24^, establishing public policies for prevention and healthcare according to cognitive performance seems to be the most viable path to the reality of countries that have not yet consolidated a sufficiently robust and organized long-term care network^25^.

In this study, the group with cognitive decline had a higher prevalence of one or more ADL limitations. This result confirms the theory of Quialheiro et al.^26^, in which cognitive changes interfere with the individual's abilities to understand and integrate the steps that characterize performance in daily activities, that is, that greater cognitive impairment is related to worse functionality. From a healthcare perspective, functional decline requires increased care and a greater number of professionals, which is not always possible to be offered by Brazilian institutions^27^. In addition to prevention protocols, it is essential to provide institutions with resources to care for populations with functional limitations. This includes training, greater financial support, coordination with rehabilitation services, and the implementation of care and care management technologies^27^.

Relative to sociodemographic profile, the study found results consistent with the literature, suggesting the feminization of old age^28^ and the predominant presence of oldest old adults in nursing homes. The findings align with those of Schmidt and Penna^29^, who found that the average age of institutionalized older adults falls between 70 and 79 years old, followed by 80 to 89 years old. Although the study by Fagundes et al.^4^ associates cognitive impairment with advancing age, the current analysis suggested that poorer MMSE scores were associated with older age.

Therefore, in terms of education, the association between higher MMSE scores and higher educational attainment confirms Mondini et al.^30^ hypothesis that education is considered as a protective factor against cognitive decline due to the preservation of cognitive reserve.

In this study, the association between the GOHAI index and the MMSE confirmed the relationship between poor oral health conditions in older adults and physical function^31^. Findings indicated that the number of remaining teeth in older adults was associated not only with oral health-related quality of life but also with decreased mental health and cognition, disability, and mortality in old age.

In this way, the positive correlations between MMSE, MNA, CC, and HGS in both hands can be explained by the critical role that adequate nutrition along with other lifestyle factors such as physical activity, sleep quality, and socialization can play in maintaining health, cognitive decline, and the prevention of cognitive decline and its progression to dementia^32^. Multicomponent exercise protocols were proposed, containing exercises to improve physical functioning (strength, endurance, balance, flexibility) and cognitive-motor skills (dual-task)^30^. However, studies in Brazil are scarce and the effectiveness of these interventions in institutionalized Brazilian older adults requires further investigation.

Combinations of unprocessed or minimally processed foods and neuroprotective nutrients, such as vegetables, fruits, and nuts, are listed as the ideal way to counteract the progression of cognitive decline. Furthermore, some studies addressing physical training and exercises reported positive effects on cognitive performance (short-term memory recovery, visuospatial skills, multiple aspects of executive functions) in institutional and residential settings for older people^33^. The positive correlation between MMSE and HGS is in line with recent studies, in a growing body of research, which associate muscular strength with cognitive status and incident dementia. Interventions aimed at increasing muscle strength, particularly among middle-aged adults, may hold promise for maintaining neurocognition^34^.

Although constipation is not a physiological consequence of aging, several age-related factors are associated with an increased prevalence of constipation, such as comorbidities, polypharmacy, reduced mobility, dietary changes, and reduced water intake^35^. According to the literature, around 70-80% of residents in LTCFs present symptoms of constipation^36^. Furthermore, cognitive decline directly influences functionality and self-care, which are relevant to factors contributing to constipation. Therefore, the hypothesis ratifies the negative correlation observed in this study between the Bristol Scale and the MMSE.

Regarding the duration of institutionalization, there was a decrease in the MMSE score according to the longer institutionalization time, demonstrating a negative correlation between the parameters. Recent studies^4^ have associated institutionalization with social and psychological isolation and cognitive changes in the older adults. Furthermore, reduced general stimuli and increased family distance may contribute to a higher prevalence of dementia in LTCFs. In this sense, institutionalization in nursing homes lacking cognitive stimulation and social activities could exacerbate functional decline^33^.

Aged people often have systemic and neurological diseases as well as various mental disorders that require pharmacological treatment. Anxiolytics, antidepressants, antiepileptics, and antipsychotics can impair cognition and alertness or affect the brainstem, favoring or causing disturbance of the oral and/or pharyngeal phases of swallowing^37^. Neuroleptic medications can result in extrapyramidal symptoms that can interfere with swallowing. In addition, administration of neuroleptics has been associated with alterations in iron status, plasma protein, high-density lipoprotein cholesterol, and triglycerides. Therefore, differences in anthropometric measurements and biochemical markers of nutritional status attributed to psychopharmacological treatment are suggested. Regarding chronic diseases, no correlation was observed between the type of disease and the MMSE, suggesting the need for more in-depth analyses and could be related to the high multimorbidity profile of participants.

It is noteworthy that the findings cannot be generalized, portraying the context of the institutions investigated. The COVID-19 pandemic made it impossible to continue the evaluations, reducing sample size and the statistical power of the analyses. Another limitation was the lack of control over the variables investigated, such as the association of neuroleptics and worse ADL performance. These associations require a clinical study and further information. Despite the scarcity of data on multidimensional assessment of institutionalized older adults in Brazil, this study highlights the importance of considering various factors influencing cognitive performance when addressing the health of this population.

Although our findings are in line with existing literature, the used of self-report questionnaire to evaluate cognitive decline and the exclusion of participants with sensory and communication disorders may have underestimated the prevalence of cognitive decline in a more comprehensive sample. Nevertheless, the results of the present study contribute to the analysis of the health conditions of institutionalized older adults correlated with cognitive decline, which can contribute to the adoption of preventive and health promotion measures.

Therefore, the need to include actions aimed at promoting self-care, independence, the provision of physical activity in the daily routine, environmental adaptation, and cognitive stimulation must be considered for these residents, aiming at greater autonomy and better quality of life. Based on these findings, the older population with cognitive decline or lower performance on the MMSE constitutes a group susceptible to negative health outcomes, which should be considered when developing health care plans and other care policies for institutionalized older people. The practical implications are significant, given the urgency of providing humanized and well-structured long-term care in response to the increasing prevalence of cognitive decline in this population.

The results of this study will help guide the creation of specific public health policies for LTCFs. Employing specialized professionals, such as physical educators, physiotherapists, nutritionists, dentists, psychologists, and physicians, tailored to the needs of each facility, is recommended. In addition, improving the training of caregivers and the infrastructure conditions guarantee the quality of life of residents in the physical, psychological, social and environmental aspects, and create an integrated environment that upholds justice, dignity, participation, respect, and autonomy for institutionalized older adults.
